# A104 ADENOMATOUS POLYPOSIS WITHIN TERTIARY GASTROENTEROLOGY PRACTICES: A RETROSPECTIVE STUDY

**DOI:** 10.1093/jcag/gwae059.104

**Published:** 2025-02-10

**Authors:** S X Jiang, P Tavakoli, M Rui Xuan Yu, M Ten-Pow, D Flegg, E Taylor, A Walia, S Pang, D Motomura, E Lam, R Enns, J Telford, W Xiong, N Shahidi

**Affiliations:** The University of British Columbia Faculty of Medicine, Vancouver, BC, Canada; StPaul’s Hospital, Division of Gastroenterology, Vancouver, BC, Canada; The University of British Columbia Faculty of Medicine, Vancouver, BC, Canada; StPaul’s Hospital, Division of Gastroenterology, Vancouver, BC, Canada; The University of British Columbia Faculty of Medicine, Vancouver, BC, Canada; StPaul’s Hospital, Division of Gastroenterology, Vancouver, BC, Canada; StPaul’s Hospital, Division of Gastroenterology, Vancouver, BC, Canada; StPaul’s Hospital, Division of Gastroenterology, Vancouver, BC, Canada; StPaul’s Hospital, Division of Gastroenterology, University of British Columbia, Vancouver, BC, Canada; StPaul’s Hospital, Division of Gastroenterology, University of British Columbia, Vancouver, BC, Canada; StPaul’s Hospital, Division of Gastroenterology, University of British Columbia, Vancouver, BC, Canada; StPaul’s Hospital, Division of Gastroenterology, University of British Columbia, Vancouver, BC, Canada; StPaul’s Hospital, Department of Pathology and Laboratory Medicine, University of British Columbia, Vancouver, BC, Canada; StPaul’s Hospital, Division of Gastroenterology, University of British Columbia, Vancouver, BC, Canada

## Abstract

**Background:**

Adenomatous polyposis (AP) is defined as ≥ 10 cumulative colorectal adenomas. Despite its clinical relevance, its prevalence, genetic implications, and optimal management remains unknown.

**Aims:**

Evaluate the prevalence and outcomes of AP within a tertiary gastroenterology referral center.

**Methods:**

Patients with at least 1 adenomatous colorectal polyp removed by 1 of 11 gastroenterologists at a tertiary referral center between 2013-2023 were screened for AP using a validated histopathology database. Patients with a known adenomatous polyposis syndrome and/or previous colorectal surgery prior to referral were excluded from analysis. All patients with AP (defined as ≥ 10 adenomatous colorectal polyps) underwent full chart review. Data were extracted for demographic and clinical outcomes.

**Results:**

Of 10,603 patients with at least 1 colorectal adenoma, 330 (3.1%) were diagnosed with AP. AP patients were diagnosed over a median of 5 years (IQR 1-9 years) and a median of 3 colonoscopies (IQR 2-4 colonoscopies). The frequencies of an advanced adenoma and colorectal cancer (CRC) were 236 (71.5%) and 23 (7.0%), respectively. 78.2% of CRC diagnosed before or concurrent to AP diagnosis. Genetic analysis was completed in 22 (6.8%) patients, with a deleterious mutation found in 10 (45.4%). 276 (83.6%) patients were managed endoscopically with 24 (7.3%) referred to surgery and 30 (9.1%) discharged due to age. 300 (90.9%) patients had 10-20 adenomas and 30 (9.1%) patients had > 20 adenomas. Patients with > 20 adenomas were more commonly referred to surgery (6, 20% vs. 18, 6%; p = 0.014). There was no difference in the frequency of CRC between groups (21, 7.0% in 10-20 adenomas and 2, 6.7% in >20 adenomas; p=0.195).

**Conclusions:**

In patients with ≥ 1 colorectal adenoma, the prevalence of AP was 3.1%, with an overall CRC risk of 7.0%. The majority of patients can be successfully managed endoscopically with further evidence needed to delineate appropriate surveillance interval recommendations.

**Funding Agencies:**

CAG

